# Constitution Building

**Published:** 1888-04-21

**Authors:** 


### THE DOCTOR'S PULPIT.

### CONSTITUTION BUILDING.

DARWIN has made plain to every capacity what striking changes in the way of growth or decay, development or retrogression, may take place in the course of uncounted ages. Many people have seen a horse, whose tail had been rendered comparatively useless by the foolish modern fashion of " docking," draw his skin into folds and wrinkles over a considerable part of the body with great energy and rapidity, in order thereby to drive away a tormenting fly. A thousand flies might attack a naked man, but by no possibility could he wrinkle and shake his skin and get rid of them in the same way. If he were bound hand and foot and deprived of the power of rolling himself on the ground, he must endure all the stings that all the flies in his neighbourhood could inflict upon him without redress. Why is it that the horse is endowed with this peculiar power whilst man is deprived of it? The horse has an appropriate muscle beneath his skin for this very purpose, but the man has not. It is probable that in ages very remote, when the man resembled the horse in many more particulars than he does now, he too was the possessor of this peculiar muscle. Ages of change and retrogression have come and gone since then, and now not one man in fifty is the owner of such a muscle, and even those exceptional individuals who can lay claim to it have it in such a shrunken and rudimentary form that it is of no use to them.

On the other hand, development has been as active, or more active, than retrogression. Whilst man, when his kinship with the horse was nearer, could probably use his skin with a large degree of freedom, his brain was an organ of a quality incomparably inferior to that of his less well-muscled descendant. The reasoning powers which then were comparatively rudimentary, are now well developed and efficient. The whole man has become, so to speak, a creature of a higher order, a being fitted for and living in an altogether loftier sphere. This result has been brought about by ages upon ages of change; but all those ages are only the sum total of the separate moments of time as it has passed.

The severest critic of Nature's operations generally allows her the possession of two distinguishing merits—" patience " and " sureness." Nature is both patient and sure, and sure because patient.

> The mills of God grind slowly,
> But they grind exceeding small.

Those things which Nature intends to endure she builds up deliberately. Jonah's gourd sprang up in a night, and it withered in a night. A Wellingtonia gigantea may be more than a hundred years in growing to its full stature, but it may live more than a thousand.

All these rather long preliminaries are intended to prepare the mind of the reader to think intelligently and profitably of what is to follow, and particularly to impress upon him the value of patient and continuous work. The subject of discourse is " Constitution Building." That must be carefully distinguished from " constitution preserving " and " constitution repairing." A man may preserve his own constitution, or the doctor may repair it for him. But the constitution which is in course of being built is that of a child or a youth. The child, at any rate, can do no building without guidance, and the youth can do very little. If any good is to be done by this sermon the argument must be addressed, like an architect's advertisement, " To Parents and Guardians."

The prime requisite for health, happiness, and success is a good constitution. If some physical philosophers had their way they would take care that everybody should at least begin life with this desideratum, for they would compel all men and women who project marriage to undergo a strict examination, after the fashion of insurance offices. There is much to be said in favour of such views; but however much there is to be " said," there is nothing at all to be " done," and so we need not waste time in useless discussion. It is, however, a matter of the utmost importance that a child be born with a sound and wholesome constitution. On this point it would be easy to bring forward an array of facts and arguments which would startle and shock many good, unworldly people. It is not too much to say that there are parents, originally strong and healthy themselves, who, as the result of bad living, transmit to their descendants evils, disabilities, possibilities of suffering and of misery, for which no regret on their part, and no advantages of other kinds which they are able to secure for their children, can ever atone. It is true as truth can be that " the sins of the parents are visited upon the children to the third and fourth generation." That is a fact against which men may rebel, but there is no more use in rebellion than there is in rebelling against Death when his hand is upon the heart. The thing to do is for men themselves, by self-denial and virtue, to avoid sin, and so escape danger. Self-indulgence is the curse, the ruin, and the destruction of young men in these times. For our part, so thoroughly do we hate and despise such self-indulgence that we have small pity for a man when the consequences fall upon himself alone. But when we see, as we have seen, child after child die, solely as the result of inherited disease, and the mother's heart wrung to distraction and despair, then have we wished that the inexorable spell of the terrible law of heredity could be broken, and that the offender might be punished altogether in his own person.

If a child begin with a good constitution the ordinary conditions of life in families of moderate means are generally sufficient to ensure advancement on the same lines. The foundation is good, and the manners and customs of Britons of average position, though not controlled by any very rigid rules, are for the most part rational, and tend to the preservation of child-health and to the development of child-energy. The constitution builder in such cases is not called upon to exercise any very special knowledge or skill.

But it often happens that a child does not get this fair start in life. One or both parents may have a poor or tainted constitution, and in that case the child cannot escape. It is possible of course to attach too much importance to inherited taints. Such taints may be very slight indeed, and only sufficient to influence the child in its feeblest, that is in its infantile stages. The elements of disease may be equivalent to *one*, whilst the elements of robustness may be equivalent to *twenty*, or a *hundred*, or a *thousand*. If the child be on the whole healthy at birth or soon after, it may be hoped and expected, circumstances being favourable, that the elements of health and vigour will increase, and along with this that the elements of disease and weakness will decrease. Under unfavourable circumstances the result will tend in an opposite direction. By favourable circumstances we mean wholesome food, healthy dwellings, pure air, and moderate exercise for mind and body. The building up of a wholesome, energetic, and wear-resisting constitution is a work requiring clear intelligence, practical wisdom, and unremitting patience. It is often done, and the mother, for it is generally the mother who accomplishes such a result, deserves as much praise and as high a reward as he who builds a noble cathedral or conducts an army to victory through a tedious and hostile country. She has her reward in the immediate success of her labours, but she may have a yet higher reward in the service which her child may render to future genertions and the world. In another article we purpose to go more into detail as to the principles and methods of constitution building.

